# Parameter-coupled state space models based on quasi-Gaussian fuzzy approximation

**DOI:** 10.1038/s41598-024-77731-w

**Published:** 2024-10-29

**Authors:** Yizhi Wang, Fengyuan Ma, Xiaomin Tian, Weina Chen, Yang Zhang, Shanshan Ge

**Affiliations:** 1 College of Intelligent Science and Control Engineering, Jinling Institute of Technology, Nanjing, 210000 China; 2 Fujian Key Laboratory of Functional Marine Sensing Materials, Minjiang University, Fuzhou, 350121 China

**Keywords:** Quasi-Gaussian Fuzzy Sets, Parameter-Coupled Models, Fuzzy Approximation, Pharmaceutical Equipment, State Space Models, Biomedical engineering, Mathematics and computing, Computer science

## Abstract

The accuracy of a fuzzy system’s approximation is closely tied to the performance of fuzzy control systems design, while this system’s interpretability depends on the description of a mechanical model using human language. This research introduces a quasi-Gaussian membership function characterized by a pair of parameters to achieve the sensitivity of a triangular membership function along with the interpretability of Gaussian membership functions. Consequently, a two-dimensional (2-D) quasi-Gaussian membership function is derived, and a method for establishing quasi-Gaussian fuzzy systems (QGFS) using a rectangular grid is proposed. After validating the approximation properties using the sine function for the one-dimensional (1-D) and 2-D QGFS, the systems are applied to approximate the depyrogenation tunnel, a significant piece of equipment in the pharmaceutical industry with various mechanical designs. Validation results indicate that the 1-D and 2-D QGFS can achieve an approximation error varying within a ± 5% range. Meanwhile, the 1-D and 2-D QGFSs are applied to mechanical models of the depyrogenation tunnel with satisfactory final approximation results. Lastly, the 2-D QGFS is capable of demonstrating an excellent description of models with coupled parameters.

## Introduction

### Background

In the industrial domain, the mathematical models of processes and equipment, among others, are frequently simplified ones, which often sacrifice object information. In the majority of circumstances, the more precise the model of a controlled object, the more conducive it is to enhancing the performance of the designed control system. Researchers have made developments in mathematical model construction in many fields^1,2^, regarding complexity modelling of ecological systems^3^, regarding environmental data collection and analysis^4^, regarding micro dynamic systems in material and molecules. However, on one hand, the ecological and environmental systems are usually involve in many parameters and vary among long time span, so that the parameters all together would balance the subtle change or fluctuation in specific parameter. Similarly, on the other hand, the mathematics within micro dynamic systems usually locate in chemical and quantum dynamics, which requires different research skills.

Nevertheless, given that industrial objects often possess such characteristics as parameter coupling, time-varying, and nonlinearity^5^, establishing the mathematical model of such complex systems has become difficult, or certain parameters in the model are challenging to express accurately by expressions. Hence, when the input and output data are known or partially known, approximation algorithms offer a preferable solution. Commonly used approximation methods include polynomial, neural network, and fuzzy approximations. In particular, the fuzzy approximation algorithm is a method for processing fuzzy data and conducting fuzzy reasoning, the main objective of which is to handle information that cannot be accurately described or fully comprehended, such as data with fuzziness or uncertainty. Accordingly, this algorithm plays an exceptionally significant role in industrial applications owing to its ability to comprehensively utilize expert experience to predict unknown information.

### Literature review

The significance of fuzzy approximation in the theoretical and practical realms of fuzzy systems cannot be overstated. Since the groundbreaking demonstration by Hao^6^ and others of the universal approximation capability of fuzzy systems—enabling the precise modeling of complex nonlinear systems—this methodology has found widespread application across diverse fields. This method’s utilization spans controller design, including such tasks as disturbance rejection control^7^ and management of complex nonlinear systems in agricultural machinery^8^, and extends to numerical computations^9^, risk assessments^10^, cost control^11^, fuzzy classification^12^, and model identification in ecosystems^13^. Fuzzy systems have been traditionally used to approximate objects with clearly defined parameters, such as inverted pendulums^14^, positions of robotic arms^15^, and water tanks^16^. However, these systems’ application to parameter-coupled state space equations remains relatively unexplored.

In the field of control system design, ensuring stability, accuracy, and response speed is paramount when utilizing fuzzy systems for approximation. Zeng^17^ and others indicated that the accuracy of fuzzy system approximations is closely tied to the focal points of membership functions, particularly the partitioning of subsets within the fuzzy system and the selection of membership functions. At present, triangular^18^ and Gaussian^19^ functions are the predominant choices for fuzzy approximation. Jiang^20^ and others expanded on this system by developing a one-dimensional fuzzy system into a two-dimensional pyramidal fuzzy system, thereby confirming the universal approximation potential of pyramidal fuzzy system. Qiu and Li^21^ observed that although triangular membership functions are highly adaptable to variations and can swiftly track the object being approximated, they lack intuitive alignment with human linguistic logic. By contrast, Gaussian functions offer better alignment with human language, thereby enhancing interpretability. However, these functions’ reduced sensitivity to data variations often leads to lower approximation accuracy compared with triangular functions.

At present, fuzzy approximation algorithms mainly consist of two research directions. The first direction is application improvement, mainly the method of selecting known membership functions and fuzzy system models as auxiliary tools to solve practical application problems. For example^22^, used the T–S type fuzzy system to approximately model the drilling subsea riser^23^. proposed spatial–temporal separation based on an object’s coupling characteristics and used the Gaussian fuzzy system to approximate and model the object based on the data set. The other direction is theoretical research. Although many research results have completed the universal analysis of the mathematical properties of different functions^24^, different dimensions^25^, and different system models^26^, the mentioned has often been tested using common data sets, and a blank space still remains for further applying the theoretical results to solve industrial problems.

### Novelty and contributions

This study uses the preceding discussion as basis to introduce a novel approach by proposing a 1–D quasi–Gaussian fuzzy membership function that can combine the strengths of the triangular and Gaussian functions. The proposed function explores the effects of adjusting key parameters on the smoothness of the curve. Thereafter, this function is extended to two dimensions, and the optimization of rules is validated by constructing a two-dimensional quasi-Gaussian fuzzy system (QGFS) to address such issues as rule explosion^27^, regional division, and approximation effectiveness. The current study uses the 1-D QGFS to model single-input single-output, and dual-input dual-output two-state scenarios of a depyrogenation tunnel. Subsequently, the two-dimensional (2-D) QGFS is applied to approximate a dual-input dual-output state-coupled model of the depyrogenation tunnel, followed by a discussion on the outcomes of the approximation.

The remainder of this paper is organized as follows. Section 1 introduces fuzzy approximation and its current research landscape. Section 2 delves into the quasi-Gaussian membership function, emerging from the triangular and Gaussian functions, discusses the effects of varying parameter values on the curve shape, and extends the concept to two dimensions. Section 3 outlines the construction of the 2-D quasi-Gaussian fuzzy system. Section 4 details the approximation system design, including regional division strategies, and substantiates its universal approximation capabilities. Section 5 briefly introduces the depyrogenation tunnel, which is widely used in pharmaceutical processes, and then applies the 1-D and 2-D quasi-Gaussian systems to approximate different mathematical models of the tunnel under varied conditions. This section also analyzes and discusses these approximations. Lastly, Sect. 6 summarizes the study and provides suggestions for future research.

## Quasi-Gaussian fuzzy sets

### Definition of 1-D quasi-Gaussian fuzzy membership function

The Gaussian membership function is commonly defined as follows:

1 $${\mu _A}(x)={e^{\left[ { - \frac{{{{(x - c)}^2}}}{{2{\sigma ^2}}}} \right]}},$$

where $c$represents the center of the Gaussian membership function and$\sigma$influences the function’s width.

Similarly, the triangular membership function is defined as follows:

2 $${\mu _A}(x)=\left\{ {\begin{array}{*{20}{c}} {\begin{array}{*{20}{c}} 0&{x \leqslant a} \\ {\frac{{x - a}}{{b - a}}}&{a \leqslant x \leqslant b} \end{array}} \\ {\begin{array}{*{20}{c}} {\frac{{c - x}}{{c - b}}}&{b \leqslant x \leqslant c} \\ 0&{x \geqslant c} \end{array}} \end{array}} \right.,$$

where a, b, and c are constants collectively shaping the triangular membership function.

This study uses these foundations as bases to introduces the following novel membership function:

3 $$\mu A(x)={e^{\frac{{ - {{\left| {x - \overline {{{x_i}}} } \right|}^\gamma}}}{\sigma }}}\;\gamma ,\sigma >0$$

The unique aspect of this function lies in the interplay of the values $\gamma$ and .$\sigma$. These parameters jointly influence the sharpness of the peak and degree of convergence at the base of the quasi-Gaussian fuzzy set. Adjusting the ratio of $\gamma$ to $\sigma$enables a conceptual transition, in which the Gaussian and triangular membership functions can be addressed as specific instances of the considerably general quasi-Gaussian membership function. An ensuing analysis will explore the range of values for $\gamma$ and $\sigma$, and the resultant shapes of the membership functions derived.

**Fig. 1:**
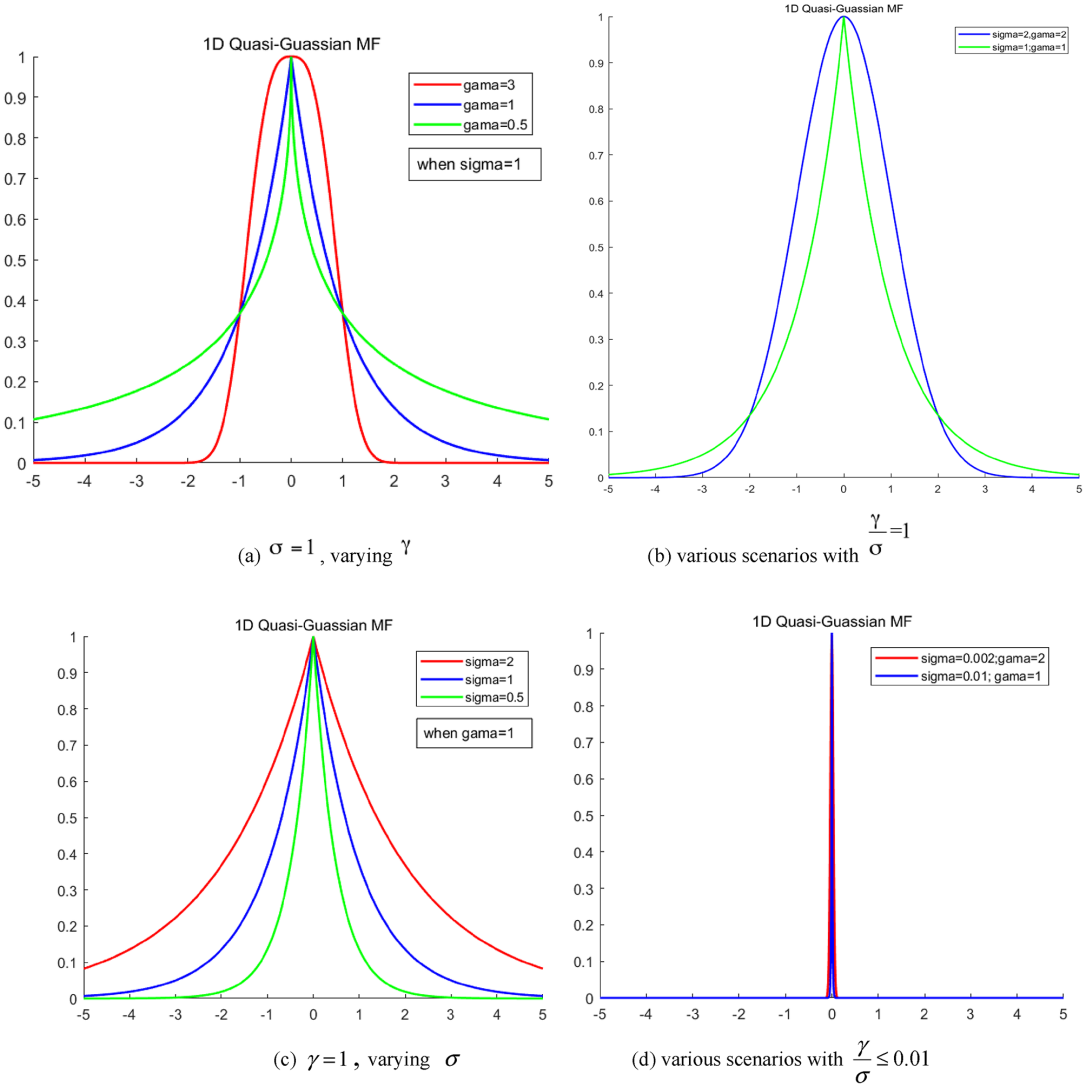
Collection of 1-D quasi-Gaussian fuzzy sets with positive parameters.

1. Maintaining a constant value for $\sigma$while varying $\gamma$ (both positive)

- When $\sigma =1$ and $\gamma >1$ (i.e., $\frac{\gamma }{\sigma }>1$), as depicted by the red curve in Fig. 1(a), the curve primarily exhibits characteristics of a Gaussian fuzzy membership function. This is marked by a smooth apex but rapid convergence toward the base, mirroring a triangular shape. As $\gamma$ increases, the membership function’s shape progressively resembles that of a trapezoid.
- When $\sigma =1$ and $\gamma =1$ (hence, $\frac{\gamma }{\sigma }=1$), as illustrated by the blue curve in Fig. 1(a) and 1(b), the curve primarily demonstrates traits of a triangular fuzzy membership function. This is characterized by a pointed apex and markedly slow convergence at the base, gradually nearing the convergence pattern of a Gaussian fuzzy membership function.
- Note that when $\sigma =2$, $\gamma =2$, equating $\frac{\gamma }{\sigma }=1$, as shown by the red curve in Fig. 1(b), the membership function closely approximates a Gaussian function. This finding suggests that the Gaussian function could be a specific instance of a quasi-Gaussian fuzzy function.
- For cases where $\sigma =1$ and $0<\gamma <1$ (thus, $0<\frac{\gamma }{\sigma }<1$), as shown by the green curve in Fig. 1(a), convergence at the apex is exceedingly swift. Within a minimal range, this could be viewed more as a conventional set than a membership set, likely leading to rapid reasoning within this scope. However, the base converges relatively slowly, closely aligning with the Gaussian fuzzy membership function.

(2) Maintaining a constant value for $\gamma$ while varying $\sigma$ (both positive)

- With $\gamma =1$, varying $\sigma$ to achieve $\frac{\gamma }{\sigma }>1$ and $0<\frac{\gamma }{\sigma }<1$, as depicted in Fig. 1(c), reveals similar trends in the top convergence curves. Each primarily reflects the convergence trend of a triangular membership function. However, as sigma decreases, the apex converges rapidly. In these scenarios, as the ratio $\frac{\gamma }{\sigma }$ increases, the curve’s overall breadth also expands, progressively leaning toward the Gaussian function’s bottom convergence pattern.

The preceding analysis indicates that when $\sigma$ and $\gamma$ are positive, $\sigma$ influences the speed of top convergence, while $\gamma$ affects the bottom convergence speed (i.e., breadth of the membership function). A smaller $\sigma$ value results in faster top convergence. When $\frac{\gamma }{\sigma } \leqslant 0.01$, the fuzzy set almost reverts to a standard set, as illustrated in Fig. 1(d).

(3) When $\sigma$is positive, $\gamma$ is negative:

**Fig. 2:**
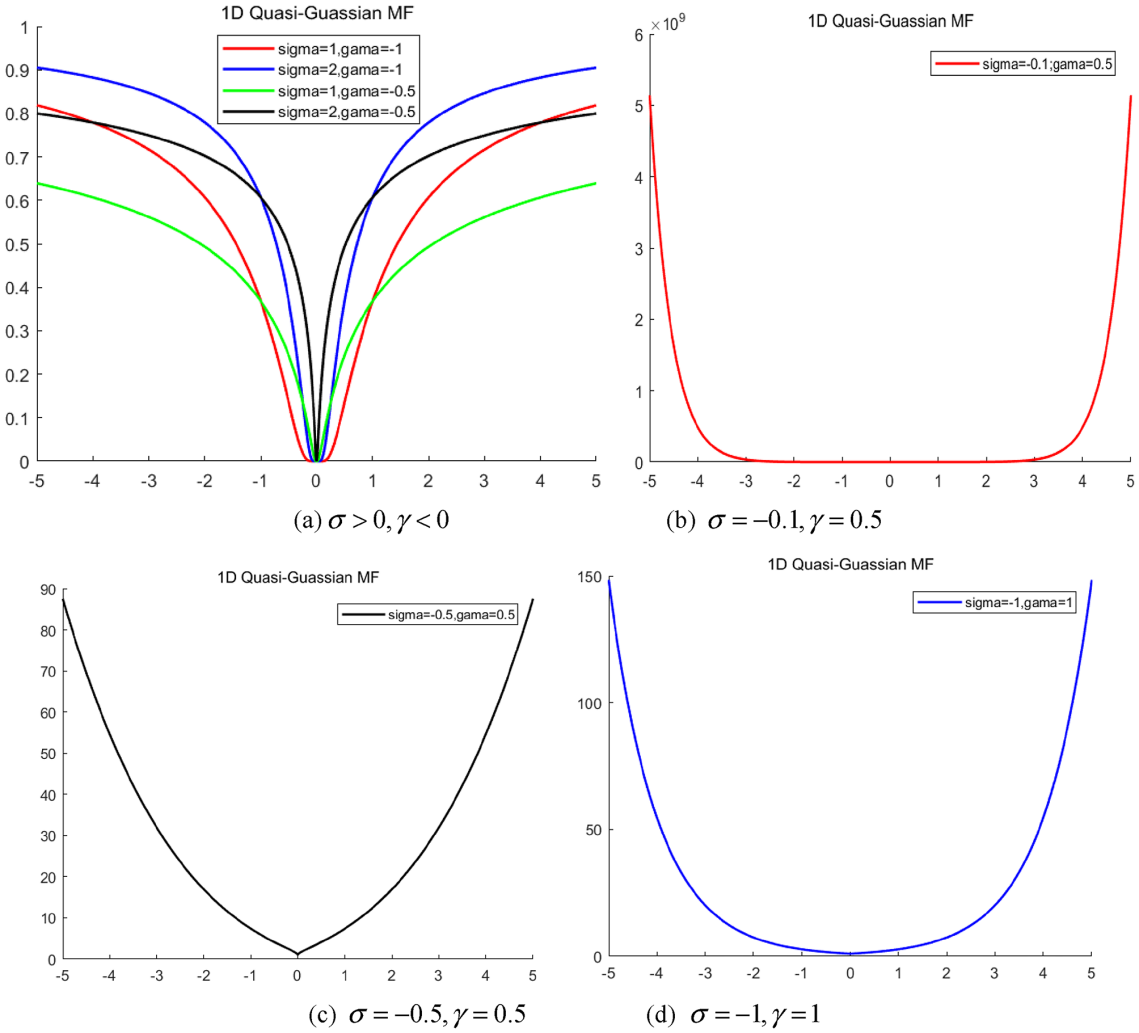
Collection of 1-D quasi-Gaussian fuzzy set collection with negative parameters.

Figure 2 (a) shows that when $\sigma$is positive and above 1 and $\gamma$is negative, the overall configuration closely mirrors that of Fig. 1 (c), flipped along the X-axis. This trend is consistent with other observations. When $\sigma$ is positive but below 1 and $\gamma$ is negative, the membership function tends to resemble an inverted trapezoidal shape (as shown in Fig. 2 (b)) and an inverted Gaussian shape (as illustrated in Fig. 2 (c)), thereby indicating divergence. Data of $\sigma$ and $\gamma$ from Fig. 2 suggest that when any parameter of the quasi-Gaussian membership function turns negative, the function transforms into a concave set. Concave fuzzy sets are rarely employed in constructing fuzzy systems owing to their divergence from common linguistic expressions and limited interpretability. Consequently, it is established that $\sigma >0,\gamma >0$ is the preferable condition.

### 2-D quasi-Gaussian fuzzy set

#### Definition 1

Let $D \subset {R^2}$ represent a bounded and closed area. Suppose $A:D \to \left[ {0,1} \right]$ is a fuzzy subset. $A$ can be considered a 2-D quasi-Gaussian fuzzy set on $D$ if it fulfills the following criteria:

(1) $$\exists ({x_0},{y_0}) \in D$$. such that $$A({x_0},{y_0})=1$$.

(2) For $\forall \lambda \in [0,1]$, the $\lambda {\text{-}}$ cut of A, denoted as ${A_\lambda }$, forms a closed subset within $D$.

**Fig. 3:**
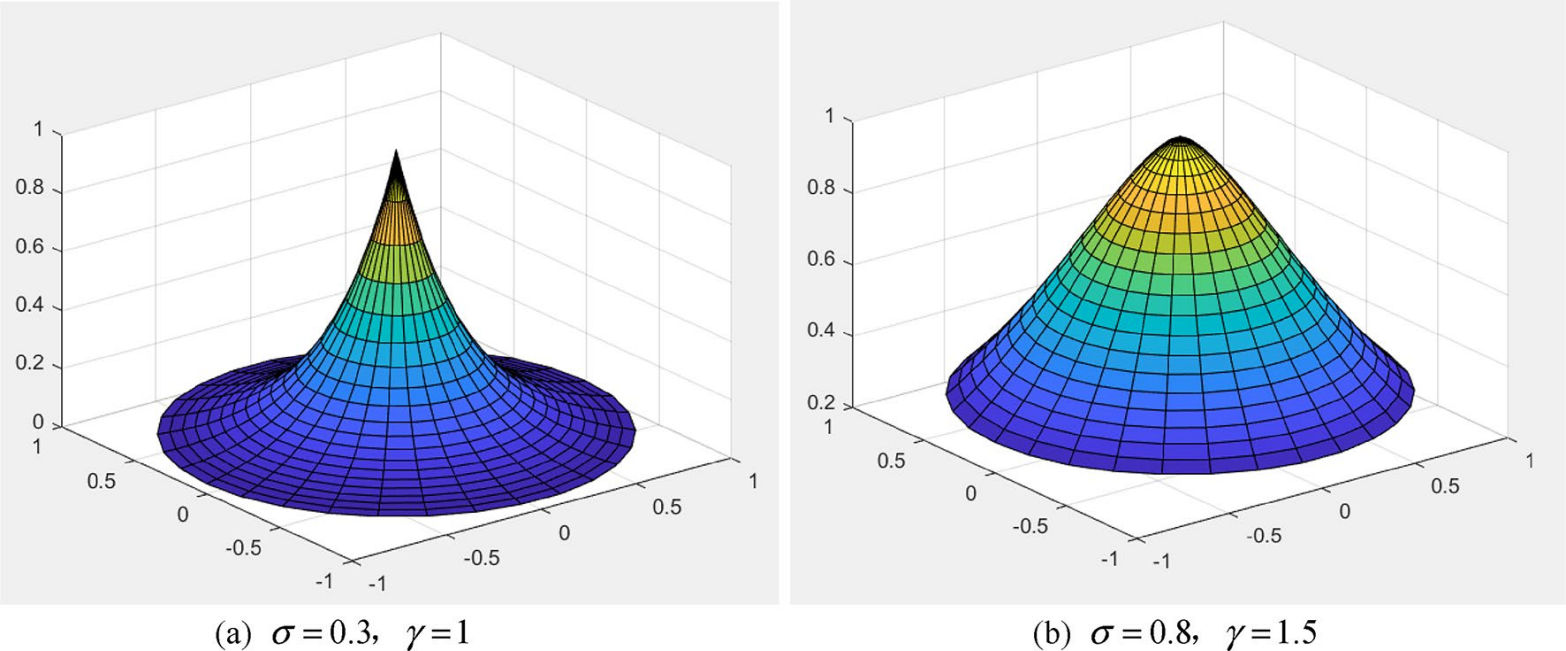
2-D quasi-Gaussian fuzzy membership function across varied $\sigma$ and $\gamma$ values

Figure 3(a) and 3(b) provide examples of quasi-type II Gaussian membership functions with various $\sigma$ and $\gamma$ values, illustrating the versatility and range of this concept.

The preceding results indicate that the parameter adjustment trends in the 2-D quasi-Gaussian fuzzy set are akin to those observed in its 1-D counterpart. This observation underscores the consistency in behavior between the two configurations of the quasi-Gaussian fuzzy set.

## Construction of the quasi-Gaussian fuzzy system

### Improvement of fuzzy rules

In traditional dual-inputs-single-output (DISO) fuzzy systems, the structure of fuzzy rules is typically as follows:

4 $${R_{ij}}:If\;x\;is\;A_{i}^{1}\;and\;y\;is\;A_{j}^{2}\;then\;z\;is\;{C_{ij}}$$

where, $i=1,2, \cdots ,M,~~j=1,2, \cdots ,N$. The antecedent of Rule (4) can be considered a fuzzy set in the input space $U \times V \subset {R^2}$, represented as $A_{i}^{1} \times A_{j}^{2}$. Its membership function is given as follows:

5 $$\left( {A_{i}^{1} \times A_{j}^{2}} \right)\left( {x,y} \right)=t\left[ {A_{i}^{i}(x),A_{j}^{2}(y)} \right],$$

where $t:[0,1] \times [0,1] \to [0,1]$ represents any t-norm operator and $A_{i}^{1}(x)$ and $A_{j}^{2}(y)$ denote the fuzzy membership functions for fuzzy sets $A_{i}^{1}$ and $A_{j}^{2}$, respectively.

This section introduces QGFS, characterized by a rule-based comprising fuzzy rules in the following format:

6 $${R_{ij}}:\;If\;(x,y)\;is\;{P_{ij}}\;then\;z\;is\;{C_{ij}},$$

where $i=1,2, \cdots ,M,~~j=1,2, \cdots ,N$; M and N are the division counts for input variables x and y, respectively; and ${P_{ij}}$ is a quasi-Gaussian fuzzy set defined on $U \times V$. Rule (6) of QGFS significantly reduces computational effort in the antecedent computation compared with conventional fuzzy system rules.

### Development of the quasi-Gaussian fuzzy system

**Fig. 4:**
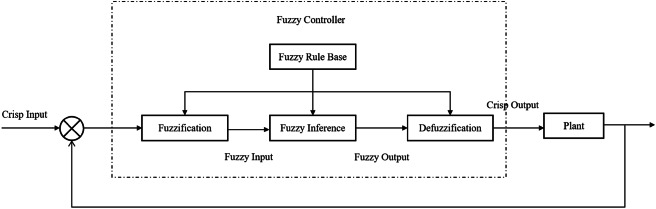
Quasi-Gaussian Fuzzy System.

As illustrated in Fig. 4, the architecture of QGFS closely resembles that of conventional fuzzy systems, consisting of four primary components: fuzzification, a fuzzy rule base, fuzzy inference, and defuzzification. The assumption is that the domains for the input variables $\left( {x,y} \right)$are $U \times V$, and the domain for the output variable z is $W$.

1. Fuzzification.

In this step, the single-point fuzzification method is utilized to transform the input data $\left( {x^{\prime},y^{\prime}} \right) \in U \times V$into a singular fuzzy set, denoted as ${P^*}$. At the data point $\left( {x^{\prime},y^{\prime}} \right)$, the membership value is assigned as 1, and it is 0 at all other points.

7 $${P^*}(x,y)=\left\{ {\begin{array}{*{20}{c}} {1,\;\left( {x,y} \right)=\left( {x^{\prime},y^{\prime}} \right)} \\ {0,\;\left( {x,y} \right) \ne \left( {x^{\prime},y^{\prime}} \right)} \end{array}} \right.$$

(2) Establishment of Fuzzy Rules.

$${R_{ij}}:~~If~\left( {x,y~} \right)~is~~{P_{ij}}~~then~~z~~is~~{C_{ij}}$$$$\left( {i=1,2, \cdots ,M,~~j=1,2, \cdots ,N} \right)$$,

where ${P_{ij}}$ represents a quasi-Gaussian fuzzy set and ${C_{ij}}$ signifies a type-1 fuzzy set. The numbers of fuzzy subsets are denoted by $M$ and $N$. When the implication operator $\theta$ is applied using the product operator, the following relation is established:

8 $${R_{ij}}\left( {x,y,z} \right)=\theta \left( {{P_{ij}}\left( {x,y} \right),{C_{ij}}(z)} \right)={P_{ij}}(x,y){C_{ij}}(z),$$

where ${P_{ij}}\left( {x,y} \right)$ and ${C_{ij}}\left( z \right)$ are the membership functions for the quasi-Gaussian fuzzy set ${P_{ij}}$ and the type-1 fuzzy set ${C_{ij}}$, respectively. Considering that the overall fuzzy relation R is as follows:

9 $$R=\mathop \cup \limits_{{i=1}}^{M} \mathop \cup \limits_{{j=1}}^{N} {R_{ij}},$$

it follows that:

10 $$R\left( {x,y,z} \right)=\mathop \vee \limits_{{i=1}}^{M} \mathop \vee \limits_{{j=1}}^{N} \left( {{P_{ij}}\left( {x,y} \right){C_{ij}}(z)} \right).$$

(3) Fuzzy Inference.

Through fuzzy inference, the fuzzy set ${C^*}={P^*} \circ R$ is derived, thereby leading to:

11 $${C^*}\left( z \right)=\mathop \vee \limits_{{\left( {x^{\prime},y^{\prime}} \right) \in X \times Y}} [{P^{\text{*}}}\left( {x^{\prime},y^{\prime}} \right) \wedge R\left( {x^{\prime},y^{\prime},z} \right)]=R(x,y,z).$$

(4) Defuzzification.

The common defuzzification methods include maximum of membership (MOM) method, bisector defuzzification method and center of gravity method. Here, the defuzzification method of center of gravity is adopted since this method can fully capture the impact of all fuzzy reasoning outcomes on the ultimate decision-making outcome. The precise output is obtained as follows:

12 $$z=\frac{{\int_{W} {z{C^*}(z)dz} }}{{\int_{W} {{C^*}(z)dz} }}=\frac{{\sum\limits_{{i=1}}^{{MN}} {{z_i}{C^*}({z_i})\Delta {z_i}} }}{{\sum\limits_{{i=1}}^{{MN}} {{C^*}({z_i})\Delta {z_i}} }}=\frac{{\sum\limits_{{i=1}}^{{MN}} {{z_i}\left[ {\mathop \vee \limits_{{i=1}}^{M} \mathop \vee \limits_{{j=1}}^{M} \left( {{P_{ij}}\left( {x,y} \right){C_{ij}}\left( z \right)} \right)} \right]\Delta {z_i}} }}{{\sum\limits_{{i=1}}^{{MN}} {\left[ {\mathop \vee \limits_{{i=1}}^{M} \mathop \vee \limits_{{j=1}}^{M} \left( {{P_{ij}}\left( {x,y} \right){C_{ij}}\left( z \right)} \right)} \right]\Delta {z_i}} }}.$$

In particular, when the fuzzy grid in the input domain $U \times V$ is divided equidistantly, Eq. (12) is simplified as follows:

13 $$z=\frac{{\sum\limits_{{i=1}}^{{MN}} {{z_i}\left[ {\mathop \vee \limits_{{i=1}}^{M} \mathop \vee \limits_{{j=1}}^{N} \left( {{P_{ij}}\left( {x,y} \right){C_{ij}}\left( z \right)} \right)} \right]} }}{{\sum\limits_{{i=1}}^{{MN}} {\left[ {\mathop \vee \limits_{{i=1}}^{M} \mathop \vee \limits_{{j=1}}^{N} \left( {{P_{ij}}\left( {x,y} \right){C_{ij}}\left( z \right)} \right)} \right]} }}.$$

If defuzzification is performed using the central average defuzzification method, then the precise output is obtained as follows:

14 $${z^*}=\frac{{\sum\limits_{{l=1}}^{{MN}} {{C^*}\left( {z_{C}^{l}} \right)z_{C}^{l}} }}{{\sum\limits_{{l=1}}^{{MN}} {{C^*}\left( {z_{C}^{l}} \right)} }}=\frac{{\sum\limits_{{l=1}}^{{MN}} {z_{C}^{l}\left[ {\mathop \vee \limits_{{i=1}}^{M} \mathop \vee \limits_{{j=1}}^{N} \left( {{P_{ij}}\left( {x,y} \right){C_{ij}}\left( {z_{C}^{l}} \right)} \right)} \right]} }}{{\sum\limits_{{l=1}}^{{MN}} {\left[ {\mathop \vee \limits_{{i=1}}^{M} \mathop \vee \limits_{{j=1}}^{N} \left( {{P_{ij}}\left( {x,y} \right){C_{ij}}\left( {z_{C}^{l}} \right)} \right)} \right]} }},$$

where $z_{C}^{l}$ represents the center of the fuzzy set ${C_{ij}}\left( z \right)$. Assuming the center of ${C_{ij}}\left( z \right)$ is denoted as $\left( {{x_i},{y_j}} \right)$ for indices $(i=1,2, \cdots ,M;$ and $j=1,2, \cdots ,N)$, for QGFS utilizing rectangular and triangular grids, outputs derived from the central average defuzzification method are observed to be equivalent to those obtained via the central defuzzification method.

(a) In the context of QGFS utilizing a rectangular grid, specifically within the range $\left[ {{x_i},{x_{i+1}}} \right] \times \left[ {{y_j},{y_{j+1}}} \right]$, the final output for a Mamdani-type QGFS is delineated, including the following specific instances:

15 $$S(x,y)=\frac{{{P_{ij}}\left( {x,y} \right){z_{ij}}+{P_{i+1,j}}\left( {x,y} \right){z_{i+1,j}}+{P_{i+1,j+1}}\left( {x,y} \right){z_{i+1,j+1}}+{P_{i,j+1}}\left( {x,y} \right){z_{i,j+1}}}}{{{P_{ij}}\left( {x,y} \right){+_{i+1,j}}\left( {x,y} \right)+{P_{i+1,j+1}}\left( {x,y} \right)+{P_{i,j+1}}\left( {x,y} \right)}}.$$

where ${z_{i1,j1}}=f\left( {{x_1},{y_1}} \right)\left( {{i_1}=i,i+1;{j_1}=j,j+1} \right)$ and $f\left( {x,y} \right)$ represent the actual model of the target system. In addition, ${P_{ij}}\left( {x,y} \right)$, ${P_{i+1,j}}\left( {x,y} \right)$, ${P_{i,j+1}}\left( {x,y} \right)$, and ${P_{i+1,j+1}}\left( {x,y} \right)$ are featured as the quasi-Gaussian membership functions at vertices at $\left( {{x_i},{y_j}} \right)$, $\left( {{x_i},{y_{j+1}}} \right)$, $\left( {{x_{i+1}},{y_{j+1}}} \right)$, and $\left( {{x_{i+1}},{y_j}} \right)$, respectively.

(b) For QGFS utilizing a triangular grid, with a network having vertices at $\left( {{x_1},{y_1}} \right)$, $\left( {{x_2},{y_2}} \right)$, and $\left( {{x_3},{y_3}} \right)$, the final output of a Mamdani-type QGFS is similarly defined, including:

16 $$S\left( {x,y} \right)=\frac{{{P_1}\left( {x,y} \right){z_1}+{P_2}\left( {x,y} \right){z_2}+{P_3}\left( {x,y} \right){z_3}}}{{{P_1}\left( {x,y} \right)+{P_2}\left( {x,y} \right)+{P_3}\left( {x,y} \right)}},$$

where ${z_1}=f\left( {{x_1},{y_1}} \right)$, ${z_2}=f\left( {{x_2},{y_2}} \right)$, and ${z_3}=f\left( {{x_3},{y_3}} \right)$ and $f\left( {x,y} \right)$ represent the actual model of the target system. The peak points of the quasi-Gaussian membership functions${P_1}\left( {x,y} \right)$, ${P_2}\left( {x,y} \right)$, and ${P_3}\left( {x,y} \right)$ are located at$\left( {{x_1},{y_1}} \right)$, $\left( {{x_2},{y_2}} \right)$, and $\left( {{x_3},{y_3}} \right)$, respectively.

## Design of quasi-Gaussian fuzzy systems

Constructing membership functions in fuzzy systems can be approached through various types and methods. The appropriate selection and effective construction of these functions are crucial to ensure the optimal performance of the fuzzy systems. Fundamental principles for developing membership functions are as follows:

(1) Utilizing convex functions,

(2) Favoring symmetric forms, and.

(3) Aligning with human linguistic patterns and minimizing inappropriate overlaps.

This section follows the guidelines and introduces quasi-Gaussian membership functions and outlines the construction of QGFS using rectangular grids. Given that QGFS can be expressed as piecewise functions, a detailed representation on a rectangular grid is provided.

### Definition of rectangular grid QGFS

**Fig. 5:**
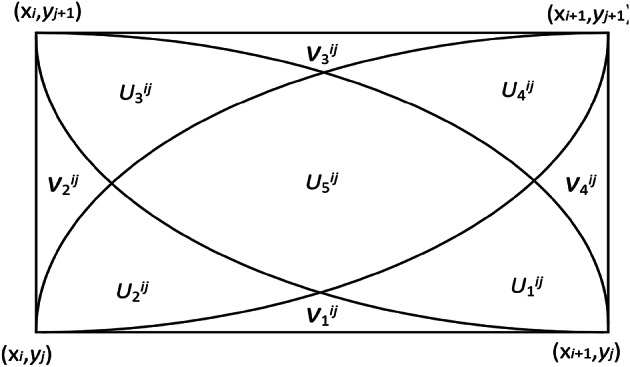
Domain division of QGFS.

In a grid defined by $\left[ {{x_i},{x_{i+1}}} \right] \times \left[ {{y_j},{y_{j+1}}} \right]$, the segmentation of the QGFS domain is illustrated in Fig. 5. This segmentation includes the delineation of areas $U_{1}^{{ij}}\sim U_{5}^{{ij}}$ and $V_{1}^{{ij}}\sim V_{4}^{{ij}}$.

where:

17 $$U_{1}^{{ij}}:y \leqslant {y_j}+{h_2}\sqrt {1 - {{(x - {x_i})}^2}/{h_1}^{2}} ;y \leqslant {y_{j+1}} - {h_2}\sqrt {1 - {{(x - {x_i})}^2}/{h_1}^{2}} ;y \geqslant {y_j}+{h_2}\sqrt {1 - {{(x - {x_{i+1}})}^2}/{h_1}^{2}}$$

18 $$U_{2}^{{ij}}:y \leqslant {y_{j+1}} - {h_2}\sqrt {1 - {{(x - {x_{i+1}})}^2}/h_{1}^{2}} ;y \leqslant {y_j}+{h_2}\sqrt {1 - {{(x - {x_{i+1}})}^2}/h_{1}^{2}} ;y \geqslant {y_j}+{h_2}\sqrt {1 - {{(x - {x_i})}^2}/h_{1}^{2}}$$

19 $$U_{3}^{{ij}}:y \leqslant {y_{j+1}} - {h_2}\sqrt {1 - {{(x - {x_i})}^2}/{h_1}^{2}} ;y \geqslant {y_j}+{h_2}\sqrt {1 - {{(x - {x_{i+1}})}^2}/{h_1}^{2}} ;y \geqslant {y_{j+1}} - {h_2}\sqrt {1 - {{(x - {x_{i+1}})}^2}/{h_1}^{2}}$$

20 $$U_{4}^{{ij}}:y \geqslant {y_{j+1}} - {h_2}\sqrt {1 - {{(x - {x_i})}^2}/{h_1}^{2}} ;y \geqslant {y_j}+{h_2}\sqrt {1 - {{(x - {x_i})}^2}/{h_1}^{2}} ;y \leqslant {y_{j+1}} - {h_2}\sqrt {1 - {{(x - {x_{i+1}})}^2}/{h_1}^{2}} .$$

21 $$\begin{gathered} U_{5}^{{ij}}:y \leqslant {y_{j+1}} - {h_2}\sqrt {1 - {{(x - {x_{i+1}})}^2}/{h_1}^{2}} ;y \leqslant {y_{j+1}} - {h_2}\sqrt {1 - {{(x - {x_i})}^2}/{h_1}^{2}} ; \hfill \\ \begin{array}{*{20}{c}} {}&{} \end{array}y \geqslant {y_j}+{h_2}\sqrt {1 - {{(x - {x_{i+1}})}^2}/{h_1}^{2}} ;y \leqslant {y_j}+{h_2}\sqrt {1 - {{(x - {x_i})}^2}/{h_1}^{2}} . \hfill \\ \end{gathered}$$

22 $$V_{1}^{{ij}}:y \leqslant {y_j}+{h_2}\sqrt {1 - {{\left( {x - {x_i}} \right)}^2}/h_{1}^{2}} ;y \leqslant {y_j}+{h_2}\sqrt {1 - {{\left( {x - {x_{i+1}}} \right)}^2}/h_{1}^{2}} .$$

23 $$V_{2}^{{ij}}:y \leqslant {y_j}+{h_2}\sqrt {1 - {{\left( {x - {x_{i+1}}} \right)}^2}/h_{1}^{2}} ;y \geqslant {y_{j+1}} - {h_2}\sqrt {1 - {{\left( {x - {x_{i+1}}} \right)}^2}/h_{1}^{2}} .$$

24 $$V_{3}^{{ij}}:y \geqslant {y_{j+1}} - {h_2}\sqrt {1 - {{\left( {x - {x_{i+1}}} \right)}^2}/h_{1}^{2}} ;y \geqslant {y_{j+1}} - {h_2}\sqrt {1 - {{\left( {x - {x_i}} \right)}^2}/h_{1}^{2}} .$$

25 $$V_{4}^{{ij}}:y \geqslant {y_{j+1}} - {h_2}\sqrt {1 - {{\left( {x - {x_i}} \right)}^2}/h_{1}^{2}} ;y \leqslant {y_j}+{h_2}\sqrt {1 - {{\left( {x - {x_i}} \right)}^2}/h_{1}^{2}} .$$

Considering $\left( {x,y} \right) \in \left[ {{x_i},{x_{i+1}}} \right] \times \left[ {{y_j},{y_{j+1}}} \right]$, and using the grid $\left[ {{x_i},{x_{i+1}}} \right] \times \left[ {{y_j},{y_{j+1}}} \right]$, the fuzzy membership functions of QGFS are characterized by peak points at four vertices, namely, $\left( {{x_i},{y_j}} \right)$, $\left( {{x_i},{y_{j+1}}} \right)$, $\left( {{x_{i+1}},{y_{j+1}}} \right)$, and $\left( {{x_{i+1}},{y_j}} \right)$, and defined as follows:

26 $${P_{ij}}(x,y)={e^{ - \frac{{{{\left( {{{\left( {x - {x_i}} \right)}^2}+{{\left( {y - {y_j}} \right)}^2}} \right)}^\gamma }}}{\sigma }}},$$

27 $${P_{i,j+1}}(x,y)={e^{ - \frac{{{{\left( {{{\left( {x - {x_i}} \right)}^2}+{{\left( {y - {y_{j+1}}} \right)}^2}} \right)}^\gamma }}}{\sigma }}},$$

28 $${P_{i+1,j}}(x,y)={e^{ - \frac{{{{\left( {{{\left( {x - {x_{i+1}}} \right)}^2}+{{\left( {y - {y_j}} \right)}^2}} \right)}^\gamma }}}{\sigma }}},\;{\text{and}}$$

29 $${P_{i+1,j+1}}(x,y)={e^{ - \frac{{{{\left( {{{\left( {x - {x_{i+1}}} \right)}^2}+{{\left( {y - {y_{j+1}}} \right)}^2}} \right)}^\gamma }}}{\sigma }}}.$$

Incorporating Equations (26) to (29) into Eq. (15), the final output of QGFS is obtained as follows:

When $$\left( {x,y} \right) \in U_{1}^{{ij}}$$

30 $$S(x,y)=\frac{{{e^{ - \frac{{{{\left( {{{\left( {x - {x_i}} \right)}^2}+{{\left( {y - {y_j}} \right)}^2}} \right)}^\gamma }}}{\sigma }}}{z_{ij}}+{e^{ - \frac{{{{\left( {{{\left( {x - {x_i}} \right)}^2}+{{\left( {y - {y_{j+1}}} \right)}^2}} \right)}^\gamma }}}{\sigma }}}{z_{i+1,j}}+{e^{ - \frac{{{{\left( {{{\left( {x - {x_{i+1}}} \right)}^2}+{{\left( {y - {y_{j+1}}} \right)}^2}} \right)}^\gamma }}}{\sigma }}}{z_{i+1,j+1}}}}{{{e^{ - \frac{{{{\left( {{{\left( {x - {x_i}} \right)}^2}+{{\left( {y - {y_j}} \right)}^2}} \right)}^\gamma }}}{\sigma }}}+{e^{ - \frac{{{{\left( {{{\left( {x - {x_i}} \right)}^2}+{{\left( {y - {y_{j+1}}} \right)}^2}} \right)}^\gamma }}}{\sigma }}}+{e^{ - \frac{{{{\left( {{{\left( {x - {x_{i+1}}} \right)}^2}+{{\left( {y - {y_{j+1}}} \right)}^2}} \right)}^\gamma }}}{\sigma }}}}}$$

When$$\left( {x,y} \right) \in U_{2}^{{ij}}$$

31 $$S(x,y)=\frac{{{e^{ - \frac{{{{\left( {{{\left( {x - {x_i}} \right)}^2}+{{\left( {y - {y_j}} \right)}^2}} \right)}^\gamma }}}{\sigma }}}{z_{ij}}+{e^{ - \frac{{{{\left( {{{\left( {x - {x_i}} \right)}^2}+{{\left( {y - {y_{j+1}}} \right)}^2}} \right)}^\gamma }}}{\sigma }}}{z_{i,j+1}}+{e^{ - \frac{{{{\left( {{{\left( {x - {x_{i+1}}} \right)}^2}+{{\left( {y - {y_j}} \right)}^2}} \right)}^\gamma }}}{\sigma }}}{z_{i+1,j}}}}{{{e^{ - \frac{{{{\left( {{{\left( {x - {x_i}} \right)}^2}+{{\left( {y - {y_j}} \right)}^2}} \right)}^\gamma }}}{\sigma }}}+{e^{ - \frac{{{{\left( {{{\left( {x - {x_i}} \right)}^2}+{{\left( {y - {y_{j+1}}} \right)}^2}} \right)}^\gamma }}}{\sigma }}}+{e^{ - \frac{{{{\left( {{{\left( {x - {x_{i+1}}} \right)}^2}+{{\left( {y - {y_j}} \right)}^2}} \right)}^\gamma }}}{\sigma }}}}}$$

When $$\left( {x,y} \right) \in U_{3}^{{ij}}$$

32 $$S(x,y)=\frac{{{e^{ - \frac{{{{\left( {{{\left( {x - {x_i}} \right)}^2}+{{\left( {y - {y_j}} \right)}^2}} \right)}^\gamma }}}{\sigma }}}{z_{ij}}+{e^{ - \frac{{{{\left( {{{\left( {x - {x_i}} \right)}^2}+{{\left( {y - {y_{j+1}}} \right)}^2}} \right)}^\gamma }}}{\sigma }}}{z_{i,j+1}}+{e^{ - \frac{{{{\left( {{{\left( {x - {x_{i+1}}} \right)}^2}+{{\left( {y - {y_{j+1}}} \right)}^2}} \right)}^\gamma }}}{\sigma }}}{z_{i+1,j+1}}}}{{{e^{ - \frac{{{{\left( {{{\left( {x - {x_i}} \right)}^2}+{{\left( {y - {y_j}} \right)}^2}} \right)}^\gamma }}}{\sigma }}}+{e^{ - \frac{{{{\left( {{{\left( {x - {x_i}} \right)}^2}+{{\left( {y - {y_{j+1}}} \right)}^2}} \right)}^\gamma }}}{\sigma }}}+{e^{ - \frac{{{{\left( {{{\left( {x - {x_{i+1}}} \right)}^2}+{{\left( {y - {y_{j+1}}} \right)}^2}} \right)}^\gamma }}}{\sigma }}}}}$$

When $$\left( {x,y} \right) \in U_{4}^{{ij}}$$

33 $$S(x,y)=\frac{{{e^{ - \frac{{{{\left( {{{\left( {x - {x_i}} \right)}^2}+{{\left( {y - {y_{j+1}}} \right)}^2}} \right)}^\gamma }}}{\sigma }}}{z_{i,j+1}}+{e^{ - \frac{{{{\left( {{{\left( {x - {x_{i+1}}} \right)}^2}+{{\left( {y - {y_j}} \right)}^2}} \right)}^\gamma }}}{\sigma }}}{z_{i+1,j}}+{e^{ - \frac{{{{\left( {{{\left( {x - {x_{i+1}}} \right)}^2}+{{\left( {y - {y_{j+1}}} \right)}^2}} \right)}^\gamma }}}{\sigma }}}{z_{i+1,j+1}}}}{{{e^{ - \frac{{{{\left( {{{\left( {x - {x_i}} \right)}^2}+{{\left( {y - {y_{j+1}}} \right)}^2}} \right)}^\gamma }}}{\sigma }}}+{e^{ - \frac{{{{\left( {{{\left( {x - {x_{i+1}}} \right)}^2}+{{\left( {y - {y_j}} \right)}^2}} \right)}^\gamma }}}{\sigma }}}+{e^{ - \frac{{{{\left( {{{\left( {x - {x_{i+1}}} \right)}^2}+{{\left( {y - {y_{j+1}}} \right)}^2}} \right)}^\gamma }}}{\sigma }}}}}$$

When $$\left( {x,y} \right) \in U_{5}^{{ij}}$$

34 $$S(x,y)=\frac{{{e^{ - \frac{{{{\left( {{{\left( {x - {x_i}} \right)}^2}+{{\left( {y - {y_j}} \right)}^2}} \right)}^\gamma }}}{\sigma }}}{z_{ij}}+{e^{ - \frac{{{{\left( {{{\left( {x - {x_i}} \right)}^2}+{{\left( {y - {y_j}} \right)}^2}} \right)}^\gamma }}}{\sigma }}}{z_{i,j+1}}+{e^{ - \frac{{{{\left( {{{\left( {x - {x_{i+1}}} \right)}^2}+{{\left( {y - {y_j}} \right)}^2}} \right)}^\gamma }}}{\sigma }}}{z_{i+1,j}}+{e^{ - \frac{{{{\left( {{{\left( {x - {x_{i+1}}} \right)}^2}+{{\left( {y - {y_{j+1}}} \right)}^2}} \right)}^\gamma }}}{\sigma }}}{z_{i+1,j+1}}}}{{{e^{ - \frac{{{{\left( {{{\left( {x - {x_i}} \right)}^2}+{{\left( {y - {y_j}} \right)}^2}} \right)}^\gamma }}}{\sigma }}}+{e^{ - \frac{{{{\left( {{{\left( {x - {x_i}} \right)}^2}+{{\left( {y - {y_j}} \right)}^2}} \right)}^\gamma }}}{\sigma }}}+{e^{ - \frac{{{{\left( {{{\left( {x - {x_{i+1}}} \right)}^2}+{{\left( {y - {y_j}} \right)}^2}} \right)}^\gamma }}}{\sigma }}}+{e^{ - \frac{{{{\left( {{{\left( {x - {x_{i+1}}} \right)}^2}+{{\left( {y - {y_{j+1}}} \right)}^2}} \right)}^\gamma }}}{\sigma }}}}}$$

When $$\left( {x,y} \right) \in V_{1}^{{ij}}$$

35 $$S(x,y)=\frac{{{e^{ - \frac{{{{\left( {{{\left( {x - {x_i}} \right)}^2}+{{\left( {y - {y_j}} \right)}^2}} \right)}^\gamma }}}{\sigma }}}{z_{ij}}+{e^{ - \frac{{{{\left( {{{\left( {x - {x_{i+1}}} \right)}^2}+{{\left( {y - {y_j}} \right)}^2}} \right)}^\gamma }}}{\sigma }}}{z_{i+1,j}}}}{{{e^{ - \frac{{{{\left( {{{\left( {x - {x_i}} \right)}^2}+{{\left( {y - {y_j}} \right)}^2}} \right)}^\gamma }}}{\sigma }}}+{e^{ - \frac{{{{\left( {{{\left( {x - {x_{i+1}}} \right)}^2}+{{\left( {y - {y_j}} \right)}^2}} \right)}^\gamma }}}{\sigma }}}}}$$

When $$\left( {x,y} \right) \in V_{2}^{{ij}}$$

36 $$S(x,y)=\frac{{{e^{ - \frac{{{{\left( {{{\left( {x - {x_i}} \right)}^2}+{{\left( {y - {y_j}} \right)}^2}} \right)}^\gamma }}}{\sigma }}}{z_{ij}}+{e^{ - \frac{{{{\left( {{{\left( {x - {x_i}} \right)}^2}+{{\left( {y - {y_{j+1}}} \right)}^2}} \right)}^\gamma }}}{\sigma }}}{z_{i,j+1}}}}{{{e^{ - \frac{{{{\left( {{{\left( {x - {x_i}} \right)}^2}+{{\left( {y - {y_j}} \right)}^2}} \right)}^\gamma }}}{\sigma }}}+{e^{ - \frac{{{{\left( {{{\left( {x - {x_i}} \right)}^2}+{{\left( {y - {y_{j+1}}} \right)}^2}} \right)}^\gamma }}}{\sigma }}}}}$$

When $$\left( {x,y} \right) \in V_{3}^{{ij}}$$

37 $$S(x,y)=\frac{{{e^{ - \frac{{{{\left( {{{\left( {x - {x_i}} \right)}^2}+{{\left( {y - {y_{j+1}}} \right)}^2}} \right)}^\gamma }}}{\sigma }}}{z_{i,j+1}}+{e^{ - \frac{{{{\left( {{{\left( {x - {x_{i+1}}} \right)}^2}+{{\left( {y - {y_{j+1}}} \right)}^2}} \right)}^\gamma }}}{\sigma }}}{z_{i+1,j+1}}}}{{{e^{ - \frac{{{{\left( {{{\left( {x - {x_i}} \right)}^2}+{{\left( {y - {y_{j+1}}} \right)}^2}} \right)}^\gamma }}}{\sigma }}}+{e^{ - \frac{{{{\left( {{{\left( {x - {x_{i+1}}} \right)}^2}+{{\left( {y - {y_{j+1}}} \right)}^2}} \right)}^\gamma }}}{\sigma }}}}}$$

When $$\left( {x,y} \right) \in V_{4}^{{ij}}$$

38 $$S(x,y)=\frac{{{e^{ - \frac{{{{\left( {{{\left( {x - {x_i}} \right)}^2}+{{\left( {y - {y_j}} \right)}^2}} \right)}^\gamma }}}{\sigma }}}{z_{ij}}+{e^{ - \frac{{{{\left( {{{\left( {x - {x_{i+1}}} \right)}^2}+{{\left( {y - {y_{j+1}}} \right)}^2}} \right)}^\gamma }}}{\sigma }}}{z_{i+1,j+1}}}}{{{e^{ - \frac{{{{\left( {{{\left( {x - {x_i}} \right)}^2}+{{\left( {y - {y_j}} \right)}^2}} \right)}^\gamma }}}{\sigma }}}+{e^{ - \frac{{{{\left( {{{\left( {x - {x_{i+1}}} \right)}^2}+{{\left( {y - {y_{j+1}}} \right)}^2}} \right)}^\gamma }}}{\sigma }}}}}$$

### Universal approximation of the QGFS system

Illustrating with QGFS using a rectangular grid, the fuzzy basis function of QGFS is defined as follows.

**Definition 2**

39 $${\varphi _{{i_1},{j_1}}}\left( {x,y} \right)=\frac{{{P_{{i_1},{j_1}}}\left( {x,y} \right)}}{{{P_{ij}}\left( {x,y} \right)+{P_{i+1,j}}\left( {x,y} \right)+{P_{i+1,j+1}}\left( {x,y} \right)+{P_{i,j+1}}\left( {x,y} \right)}}$$

where ${i_1}=(i,i+1)$ and ${j_1}=(j,j+1)$, and ${P_{{i_1},{j_1}}}\left( {x,y} \right)$is a quasi-Gaussian membership function.

According to Definition 2, QGFS defined in a rectangular grid is expressed as follows:

40 $${S_1}\left( {x,y} \right)={\varphi _{i,j}}\left( {x,y} \right){z_{i,j}}+{\varphi _{i+1,j}}\left( {x,y} \right){z_{i+1,j}}+{\varphi _{i+1,j+1}}\left( {x,y} \right){z_{i+1,j+1}}+{\varphi _{i,j+1}}\left( {x,y} \right){z_{i,j+1}},$$

where the functions ${\varphi _{i,j}}\left( {x,y} \right)$, ${\varphi _{i+1,j}}\left( {x,y} \right)$, ${\varphi _{i+1,j+1}}\left( {x,y} \right)$, and ${\varphi _{i,j+1}}\left( {x,y} \right)$ are assigned a value of 1 at specific points $\left( {{x_i},{y_j}} \right)$, $\left( {{x_i},{y_{j+1}}} \right)$, $\left( {{x_{i+1}},{y_{j+1}}} \right)$, and $\left( {{x_{i+1}},{y_j}} \right)$, respectively, while being valued at 0 at all other vertices of the grid. Consequently, the QGFS functions play the role of an interpolating mechanism, with its fuzzy basis functions undertaking the role of interpolating basis functions. The fuzzy basis functions are characterized by the following attributes:

41 $${\varphi _{i,j}}\left( {x,y} \right)+{\varphi _{i+1,j}}\left( {x,y} \right)+{\varphi _{i+1,j+1}}\left( {x,y} \right)+{\varphi _{i,j+1}}\left( {x,y} \right)=1.$$

That is, the fuzzy basis functions in QGFS serve dual roles as weighting functions. Consequently, the QGFS output can be interpreted as a weighted summation of the values of the fuzzy basis functions.

#### Theorem 1

For any continuous real function $f\left( {x,y} \right)$ defined on a compact set $U \times V \subset {R^2}$, a quasi-Gaussian fuzzy system $S\left( {x,y} \right)$ exists, thereby satisfying the condition ${\left\| {S - f} \right\|_\infty }<\varepsilon$.

In particular, $${\left\| {S - f} \right\|_\infty }={\sup _{\left( {x,y} \right) \in U \times V}}\left| {S\left( {x,y} \right) - f\left( {x,y} \right)} \right|$$

#### Proof

For the purposes of this demonstration, assume, without loss of generality, that a QGFS is defined on a grid $$\left[ {{x_i},{x_{i+1}}} \right] \times \left[ {{y_j},{y_{j+1}}} \right]$$with coordinates $$\forall \left( {x,y} \right) \in \left[ {{x_i},{x_{i+1}}} \right] \times \left[ {{y_j},{y_{j+1}}} \right]$$$$\left( {i=1,2, \cdots ,M,~~j=1,2, \cdots ,N} \right)$$ in the $$U \times V$$ space. Through the known interpolation characteristics of QGFS, we can deduce the following properties:

$$\left| {S\left( {x,y} \right) - f\left( {x,y} \right)} \right|=\left| {S\left( {x,y} \right) - S\left( {{x_i},{y_j}} \right)+f\left( {{x_i},{y_j}} \right) - f\left( {x,y} \right)} \right|$$

$$\leqslant \left| {S\left( {x,y} \right) - S\left( {{x_i},{y_j}} \right)} \right|+\left| {f\left( {{x_i},{y_j}} \right) - f\left( {x,y} \right)} \right|$$

By invoking the Lagrange mean value theorem, it can be demonstrated that there exist points $\left( {{\xi _1},{\eta _1}} \right)$ and $\left( {{\xi _2},{\eta _2}} \right)$ within the domain $\left( {{x_i},{x_{i+1}}} \right) \times \left( {{y_j},{y_{j+1}}} \right)$, such that:

42 $$\begin{gathered} \left| {S\left( {x,y} \right) - f\left( {x,y} \right)} \right| \hfill \\ \leqslant \left| {{{\left. {\frac{{\partial S}}{{\partial x}}} \right|}_{({\xi _1},{\eta _1})}}\left( {x - {x_i}} \right)+{{\left. {\frac{{\partial S}}{{\partial y}}} \right|}_{({\xi _1},{\eta _1})}}\left( {y - {y_j}} \right)} \right|+\left| {{{\left. {\frac{{\partial f}}{{\partial x}}} \right|}_{({\xi _2},{\eta _2})}}\left( {x - {x_i}} \right)+{{\left. {\frac{{\partial f}}{{\partial y}}} \right|}_{({\xi _2},{\eta _2})}}\left( {y - {y_j}} \right)} \right| \hfill \\ \leqslant \left( {\left| {{{\left. {\frac{{\partial S}}{{\partial x}}} \right|}_{({\xi _1},{\eta _1})}}} \right|+\left| {{{\left. {\frac{{\partial S}}{{\partial y}}} \right|}_{({\xi _1},{\eta _1})}}} \right|+\left| {{{\left. {\frac{{\partial f}}{{\partial x}}} \right|}_{({\xi _2},{\eta _2})}}} \right|+\left| {{{\left. {\frac{{\partial f}}{{\partial y}}} \right|}_{({\xi _2},{\eta _2})}}} \right|} \right)h \hfill \\ \end{gathered}$$

where $$h=\hbox{max} \left\{ {\left| {x - {x_i}} \right|,\left| {y - {y_j}} \right|} \right\}$$.

Given that $\left( {\left| {{{\left. {\frac{{\partial S}}{{\partial x}}} \right|}_{({\xi _1},{\eta _1})}}} \right|+\left| {{{\left. {\frac{{\partial S}}{{\partial y}}} \right|}_{({\xi _1},{\eta _1})}}} \right|+\left| {{{\left. {\frac{{\partial f}}{{\partial x}}} \right|}_{({\xi _2},{\eta _2})}}} \right|+\left| {{{\left. {\frac{{\partial f}}{{\partial y}}} \right|}_{({\xi _2},{\eta _2})}}} \right|} \right)$ is a constant, it follows that when h is sufficiently small, the following $\forall \varepsilon >0$ and $\forall (x,y) \in U \times V$ are valid, such that:

43 $$\left\{ \begin{gathered} \left| {S\left( {x,y} \right) - f\left( {x,y} \right)} \right|<\varepsilon \hfill \\ {\left\| {S - f} \right\|_\infty }={\sup _{\left( {x,y} \right) \in U \times V}}\left| {S\left( {x,y} \right) - f\left( {x,y} \right)} \right|<\varepsilon \hfill \\ \end{gathered} \right.$$

## Approximation results and analysis

### Approximation with one-dimensional QDFS

**Fig. 6:**
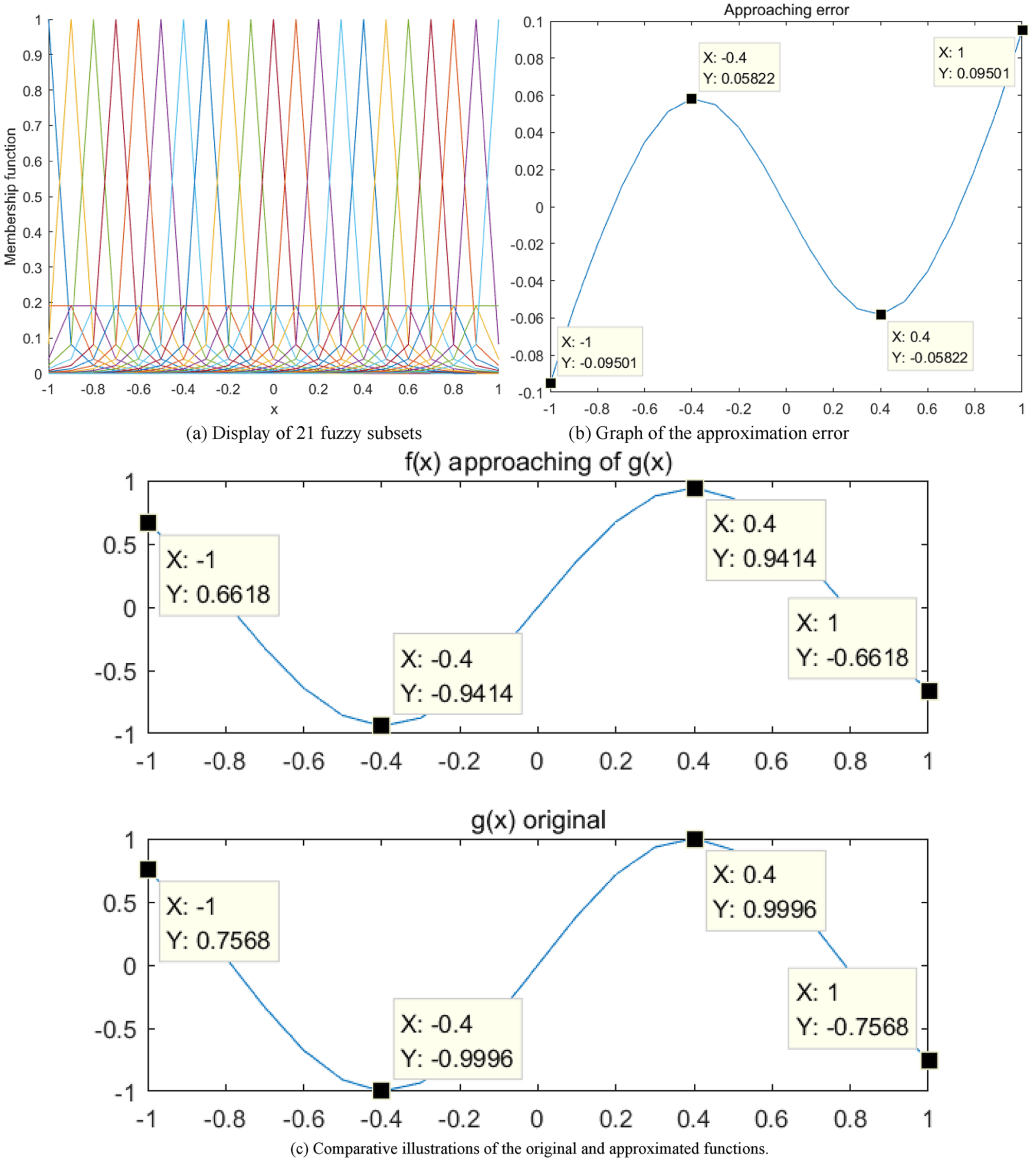
Approximation results of $y=\sin (x)$ using a 1-D QGFS

In this study, $y=\sin (x)$ is approximated using a 1-D Gaussian fuzzy system. Parameters for the input variable ${x_1}$are set at $\sigma =0.1,\gamma =0.6$. The interval $\left[ { - 1,1} \right]$is partitioned into fuzzy subsets at a step size of 0.05, yielding a total of 21 subsets. These subsets are depicted in Fig. 6(a). Thereafter, this fuzzy system approximates the sine function $y=\sin (x)$. The resulting approximation error is illustrated in Fig. 6(b), varying within the range of ± 5%. The curve of the approximation is presented in Fig. 6(c).

### Approximation with 2-D QDFS

**Fig. 7:**
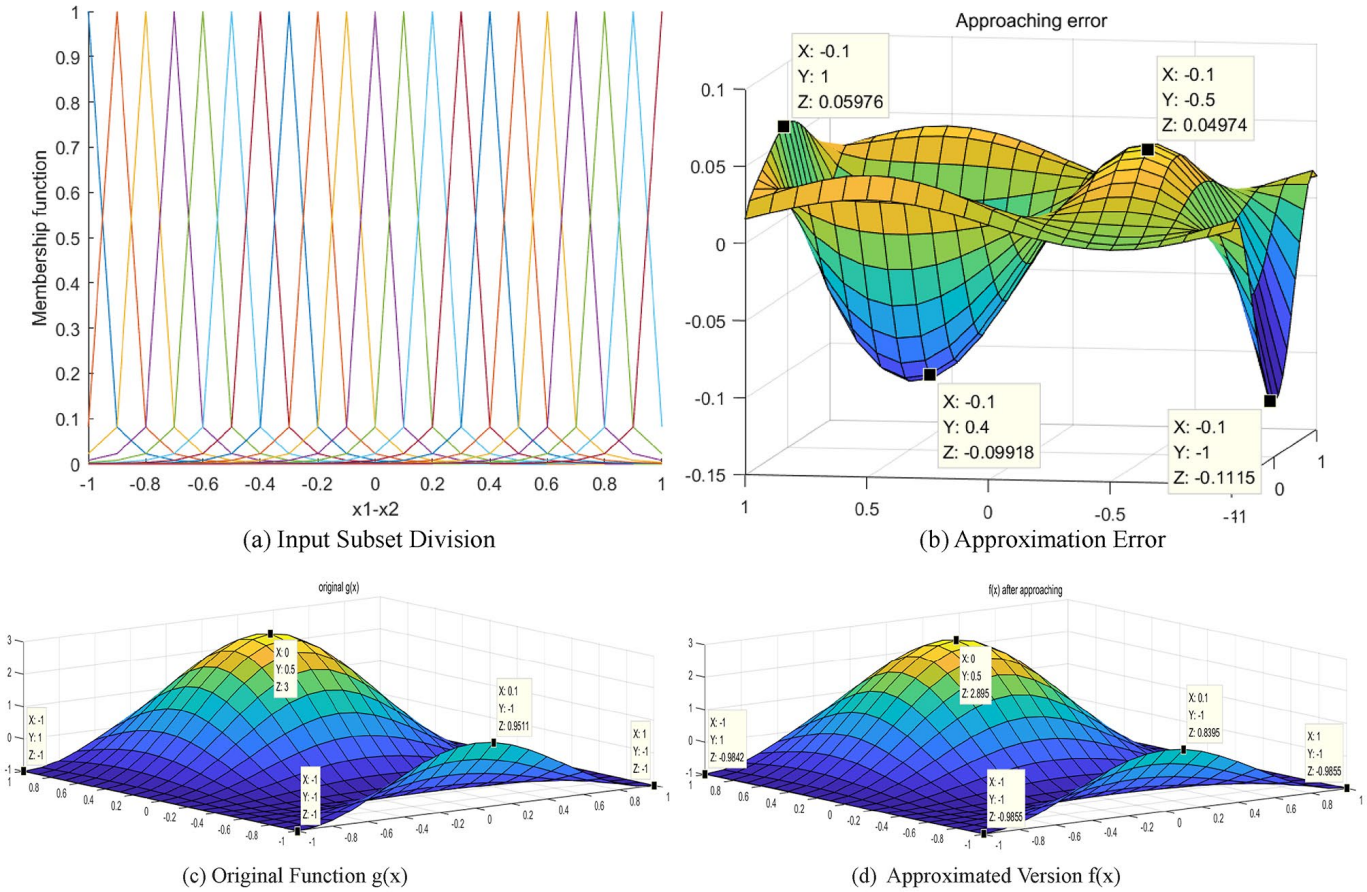
Results of approximation using 2-D QGFS.

In a further investigation, $y=\sin (x)$is approximated using a 2-D QGFS. For this approximation, the parameters $\sigma =0.1,\gamma =0.6$ are assigned to input variables ${x_1}$ and ${x_2}$. The interval [− 1, 1] is again divided into fuzzy subsets with a step size of 0.05. The result is 21 subsets for each variable, cumulatively forming $21 \times 21=441$ rules. The configuration of the rules is presented in Fig. 7(a). The approaching error is shown in Fig. 7(b), with an error range of [− 0.15,0.05]. Lastly, the approaching results are shown in Fig. 7(c) and (d).

**Fig. 8:**
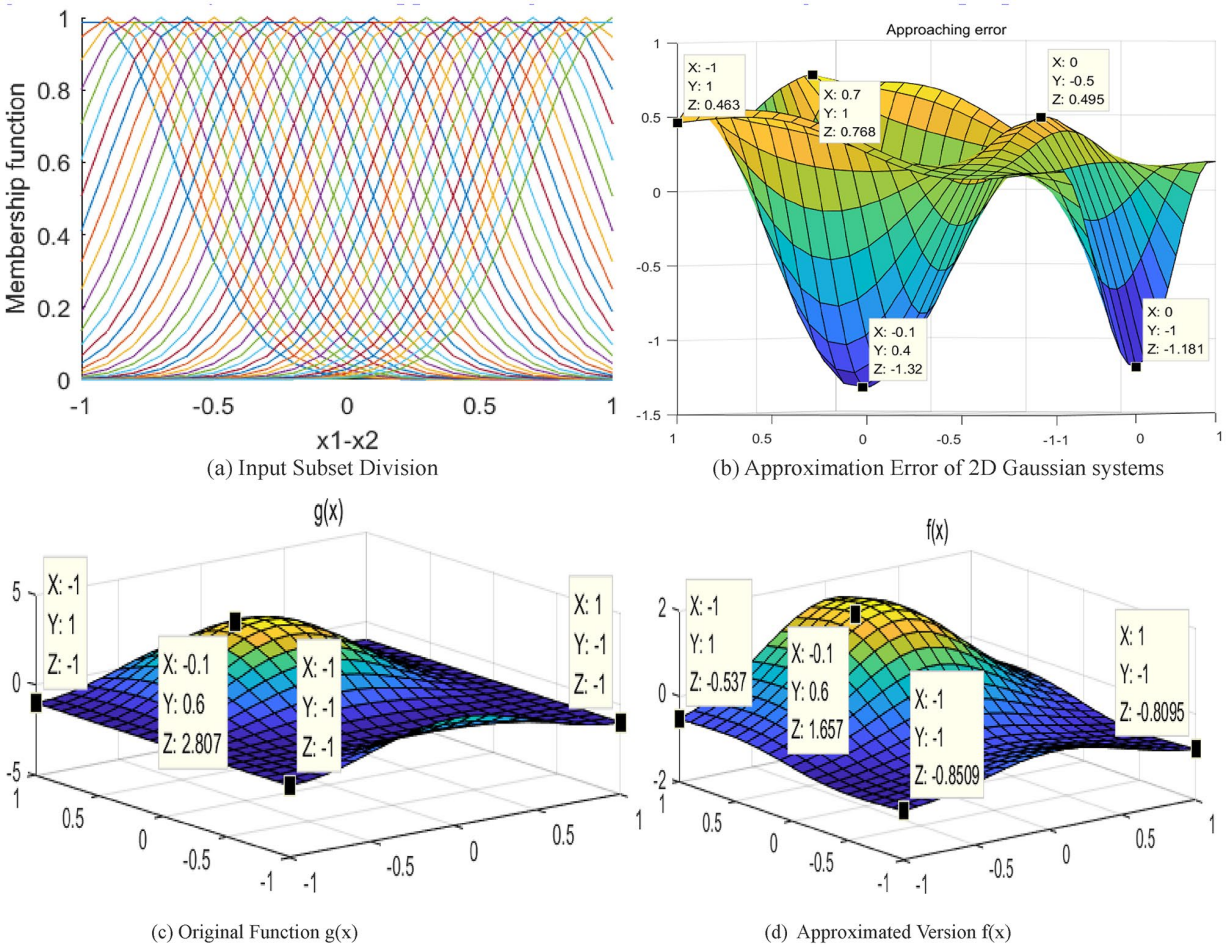
Results of approximation using 2-D Gaussian fuzzy system.

To demonstrate the effectiveness of the proposed method, the 2-D Gaussian type fuzzy approximation system is applied to the same sine function, with $\sigma =0.3$. The results are given in Fig. 8. Evidently, the overall approaching error is 10 times larger than the proposed method.

### QDFS approaching with a case study

Regarding industrial equipment and process improvement, there are many excellent research results, such as the equipment mechanical structure improvement^28^ and the process improvement in^29^. However, as neither the mechanical structure modification nor the process improvement could possibly be implemented before an extreme long term of equipment qualification or process validation, the modelling and approximation approach gives a solution to obtain an accurate mathematical model for follow-up control system design.

The depyrogenation tunnel stands as an essential apparatus within the pharmaceutical field, particularly in the production of formulations. Its principal role involves the ongoing sterilization and cleansing of packaging materials, such as vials and ampoules. Furthermore, it serves a critical function in the drying of specific active pharmaceutical ingredient (API) powders^30^. Given that the ultimate products are directly administered to the human body, the efficacy of sterilization holds the utmost importance. Consequently, temperature regulation emerges as a pivotal process parameter, necessitating vigilant monitoring of lower temperature thresholds and ensuring consistent temperature control. Moreover, given that the depyrogenation tunnels are operated continuously rather than in batches, the management of internal wind pressure becomes imperative. This management is essential to prevent cross-contamination between processes stemming from unpredictable pressure fluctuations. Hence, precise and timely control of wind pressure stands as a pivotal process parameter. Wang^31,32^ and others previously conducted numerical simulations to investigate the internal temperature and airflow patterns within the depyrogenation tunnel utilized for vial sterilization. Their study included modeling the state space for wind pressure and temperature, implementing control strategies using pole placement, and utilizing fuzzy logic and PID control systems. In addition, they delved into verification processes for equipment employed in drying APIs. This section builds upon the findings, parameters, and models outlined in their study to approximate diverse modeling scenarios using 1-D and 2-D QDFS. Subsequently, the results of these approximations are meticulously scrutinized and analyzed.

Data Reference as given in Table 1:

**Table 1:** Equipment Parameters for the Continuous Drying of Vials^33^.

Parameters	Values
*Ρ*	0.615 kg/m^3^ (at 300 °C)
*M*_*o*_	2.4 kg (5 ml Vials*1500 pcs for one batch)
*C*_*a*_	1.0 × 10^3^ J/ (kg. °C)
*C*_*o*_	9.66 × 10^2^ J/ (kg °C)
*T*_*0(a)*_	350 °C (specified)
*T*_*t(a)*_	320 °C (Ideally)
*T*_*0(o)*_	280 °C
*T*_*t(o)*_	320 °C (Required)
*d*	0.3 m

By integrating the specified parameters into the state space Eq. (44) as referenced from^34^:

44 $$\left\{ \begin{gathered} \mathop X\limits^{\cdot } =\left[ {\begin{array}{*{20}{c}} { - \rho }&0 \\ {\frac{{{C_a}\rho (1 - \rho ){u_2}}}{{{C_o}{M_o}}}}&0 \end{array}} \right]\left[ {\begin{array}{*{20}{c}} {{x_1}} \\ {{x_2}} \end{array}} \right]+\left[ {\begin{array}{*{20}{c}} {\frac{1}{S}}&0 \\ 0&{\frac{{{C_a}\rho {u_1}}}{{{C_o}{M_o}S}}} \end{array}} \right]\left[ {\begin{array}{*{20}{c}} {{u_1}} \\ {{u_2}} \end{array}} \right] \hfill \\ \mathop Y\limits^{\cdot } =\left[ {\begin{array}{*{20}{c}} 1&0 \\ 0&1 \end{array}} \right]\left[ {\begin{array}{*{20}{c}} {{x_1}} \\ {{x_2}} \end{array}} \right]+D\left[ {\begin{array}{*{20}{c}} {{u_1}} \\ {{u_2}} \end{array}} \right] \hfill \\ \end{gathered} \right.,$$

where ${u_1}={Q_a},{u_2}=\Delta {T_a}$ represent the variations in heat flux and the changes in the temperature of dry hot air, respectively, when hot air pressure changes. Depending on the specific case, the QDFS method is utilized to approximate the outputs of the state space equations previously described.

1. 1-D QGFS approximation for the SISO state-space model.

**Fig. 9:**
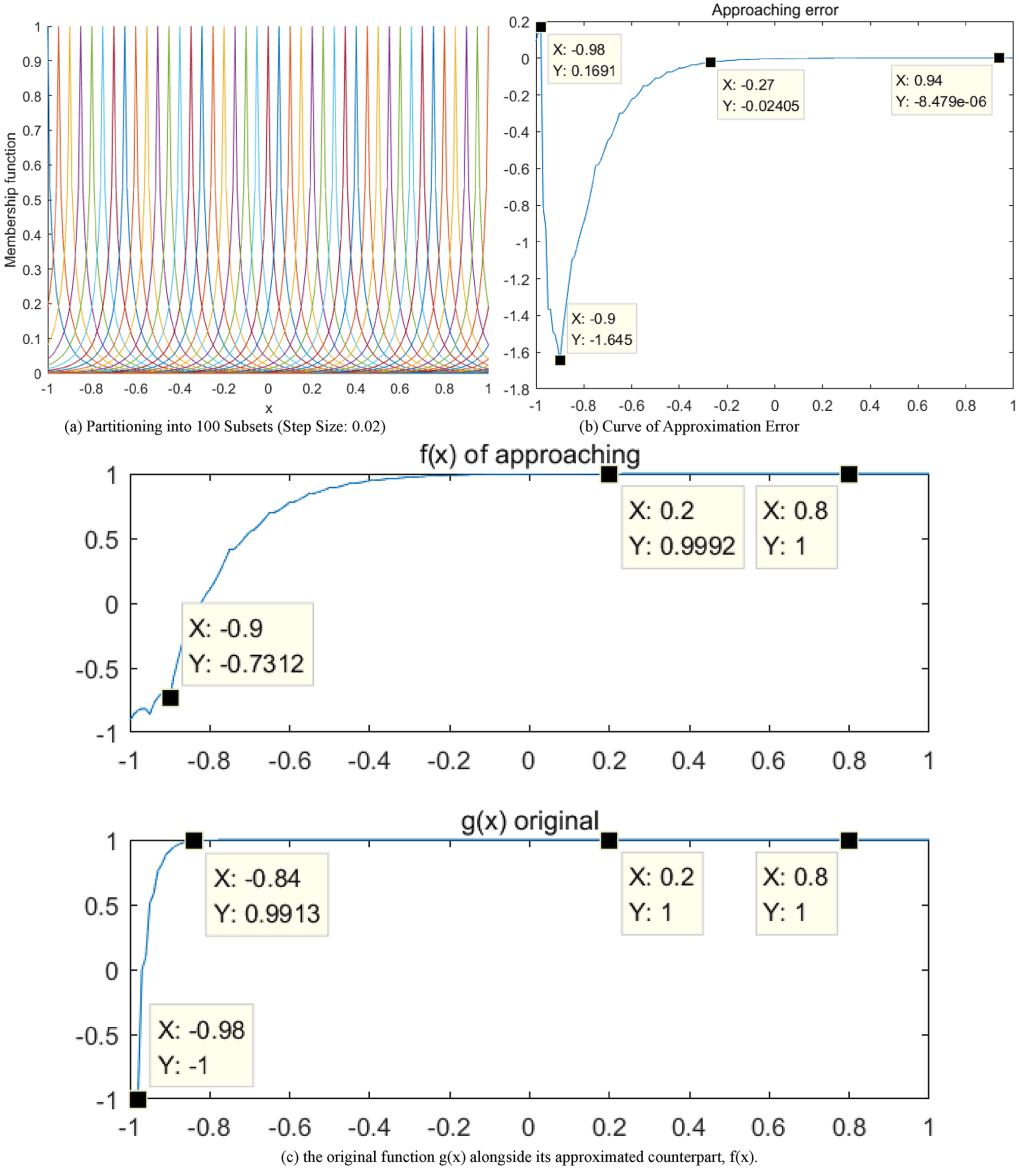
Approximation results of the state-space model with single state variable using one-dimensional QDFS.

In the case of a single state variable (with the setting of ${x_2}=0$), 100 fuzzy subsets were created within the range of [− 1,1] and with a step size of 0.02. Parameters for the quasi-Gaussian membership function were set to $\sigma =0.1,\gamma =0.6$. This setup is used for approximating the output of Eq. (44). The results of this approximation are illustrated in Fig. 9. Specifically, at the initial several steps the approximation error was quite large, which is probably due to prominent effect of the Gaussian-like non-sensitive lower part of fuzzy subsets at the beginning.

(2) 1-D QGFS approximation for the two-input-two-output state-space model.

**Fig. 10:**
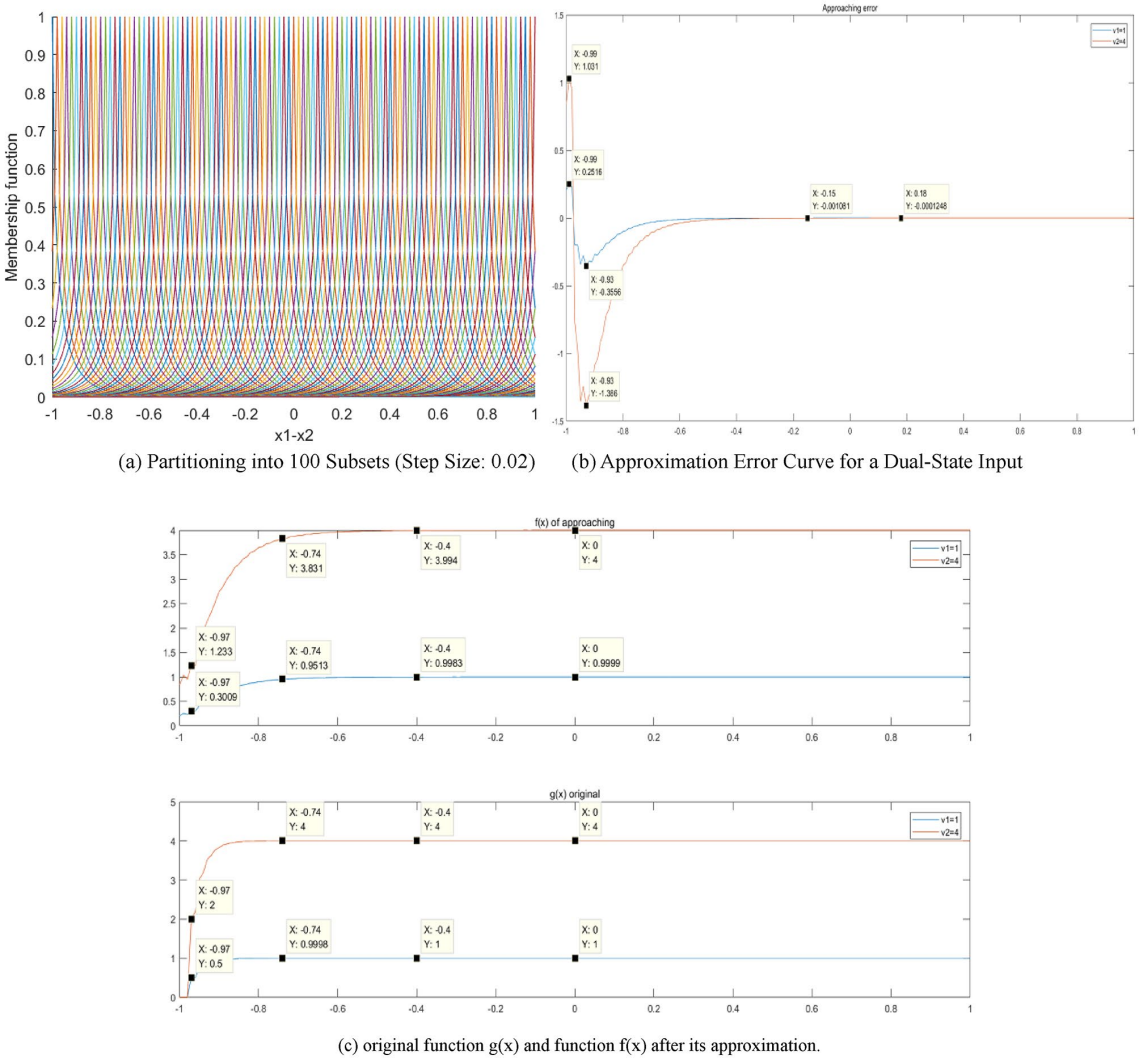
Approximating results of dual-state variable model using one-dimensional QGFS.

In certain devices, hot air pressure data are gathered for monitoring purposes (as a state variable), yet it is not employed as a control variable. In such scenarios, the constructed state-space model incorporates two state variables, resulting in a system with dual outputs. Owing to the non-utilization of this parameter as a control variable, the two-state variables operate independently, without any interconnection. Consequently, the state variable x_2_, which represents hot air pressure, is considered for approximation using 1-D QDFS. A total of 100 fuzzy subsets were created within the range of [− 1,1] and with a step size of 0.02. Parameters for the quasi-Gaussian membership function were set to $\sigma =0.1,\gamma =0.6$ to approximate Eq. 44. In this context, the object’s two states are treated as independent (i.e., without any coupling). The approximation results are shown in Fig. 10.

(3) 2-D QDFS Approaching Considering Parameter Coupling.

**Fig. 11:**
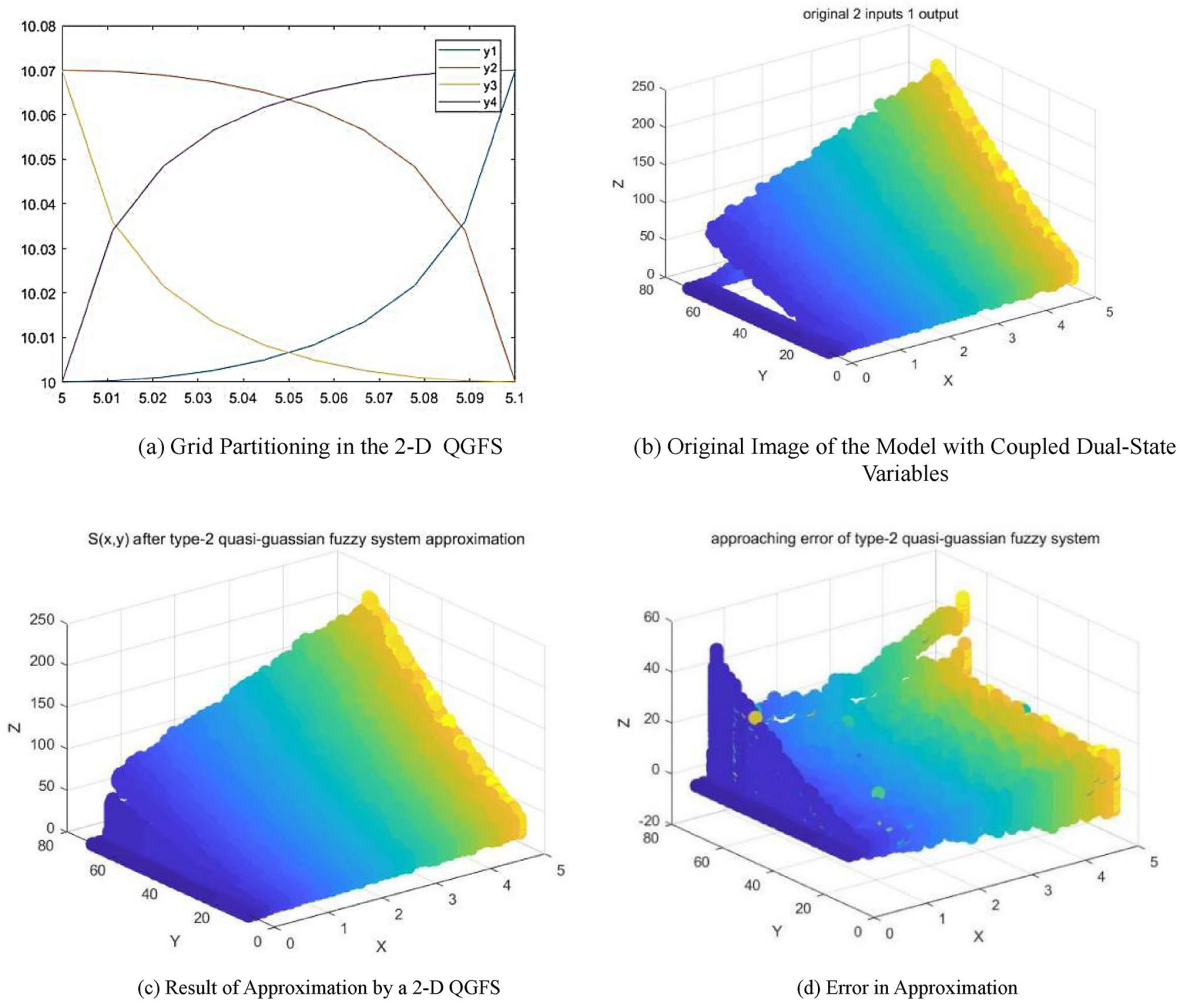
Results of the approximation by a 2-D QGFS for a model with coupled dual-state variables.

Incorporating wind pressure control transforms the model into a system with two inputs, two outputs, and two states. Wind pressure control, realized through the conduit supplying hot air from a tunnel, inherently affects temperature. Thus, adjusting wind pressure not only controls the wind but also alters the temperature, creating a coupling effect between the two states. To address this, we apply a two-dimensional QDFS for the approximation.100 fuzzy subsets were created within the range of [-1,1] and with a step size of 0.02. The parameters for the quasi-Gaussian membership function were set to $\sigma =0.1,\gamma =0.6$. This model utilizes a rectangular grid to facilitate the approximation of Eq. (44). The segmentation of intervals is depicted in Fig. 11(a). The original curve is shown in Fig. 11(b). The curve representing the approximation is shown in Fig. 11(c). Lastly, the approximation error is detailed in Fig. 11(d).

The presented results highlight that upon integrating real-world models and parameters and introducing step disturbances, the 1-D and 2-D QGFS initially exhibit notable errors and a delay in data processing. However, they rapidly converge with the original data, thereby demonstrating reduced errors over time. This improvement can be attributed to the initial approximation stages primarily involving the lower, smoother section of the quasi-Gaussian function, in which sensitivity is diminished owing to the function’s gradual slope. As the process progresses, the upper, more responsive portion of the quasi-Gaussian function, resembling the triangular membership function, begins to dominate, thereby providing heightened sensitivity and improved approximation results. Although the 1-D QDFS displays faster responsiveness in simpler systems, the 2-D QDFS proves more effective in complex systems, particularly those with interconnected states and variables, offering a more nuanced representation of the interdependencies.

## Conclusions and future work

### Conclusions

Fuzzy approximation systems rely on a single parameter face limitations, particularly in their ability to effectively capture the complexities inherent in systems with interconnected parameters. To address this challenge, researchers developed a quasi-Gaussian fuzzy membership function with adjustable parameters and investigated its impact on the function’s shape. Thereafter, the aforementioned study expanded this function into a 2-D form, utilizing rectangular and triangular grids for construction, thereby confirming its broad applicability in approximating complex systems. In a practical demonstration, 1-D and 2-D QGFS were applied to approximate the mathematical model of a depyrogenation tunnel—a crucial apparatus in the pharmaceutical industry known for its parameter interdependencies owing to diverse mechanical designs. The results of the approximation show that although the 1-D QGFS achieves nearly perfect accuracy once system data stabilize, the 2-D system excels in determining the intricate parameter couplings characteristic of complex systems.

### Future research

This study introduces 1-D quasi-Gaussian fuzzy membership functions and extends them to 2-D quasi-Gaussian fuzzy basis functions, demonstrating their wide-ranging approximation capabilities. Meanwhile, the following research opportunities are identified further.

1. This research acknowledges that adjusting parameters does not fully address the issue of significant initial errors when approximating real-world models. This finding could be attributed to the method of dividing fuzzy subsets into uniform intervals, which works well for gradually varying functions (e.g., sine waves) but may be less effective for real-world models with boundary ranges that could reduce the fuzzy system’s sensitivity caused by inappropriate step sizes. Further investigation into fuzzy systems with changeable domains is warranted.
2. The utilization of 2-D QGFS for approximating complex, multi-input-multi-output systems with interconnected parameters shows promise. Despite successful dimension reduction through antecedent optimization, which simplifies computational demands, the process remains time-intensive, thereby posing challenges for real-time applications. Therefore, future research should prioritize enhancing the real-time approximation efficiency of 2-D QGFS.
3. In this study, the proposed 1-D and 2-D QGFS are used to approximate the object based on the data-driven + mechanism model, and the first-order difference method is selected for approximation. This method can obtain a definite numerical solution, but its robustness may be poor. Therefore, we can choose the method of multi-order approximation to obtain the uncertain numerical solution (value range) at some special data points to convert the precise numerical solution into the uncertain solution in a certain range.

## Data Availability

All data generated or analysed during this study are included in this published article.
